# Exploring views and experiences of childbirth-related perineal trauma: a qualitative study protocol for developing a wound management tool and care pathway

**DOI:** 10.1136/bmjopen-2024-088248

**Published:** 2025-04-25

**Authors:** Laura Jones, Amy Delicate, Susan Waigwa, Victoria Hodgetts Morton, R Katie Morris, Jane Whitehurst, Sarah Hillman, Chapter Collaborative Group, Sara Webb

**Affiliations:** 1 Department of Applied Health Sciences, School of Health Sciences, College of Medicine and Health, University of Birmingham, Birmingham, UK; 2 Department of English Language and Linguistics, School of English, Drama and Creative Studies, College of Arts and Law, University of Birmingham, Birmingham, UK; 3 Birmingham Women’s Hospital, Birmingham, UK; 4 NIHR Applied Collaboration West Midlands, Birmingham, UK; 5 Warwick Medical School, University of Warwick, Coventry, UK

**Keywords:** OBSTETRICS, Postpartum Women, Natural Childbirth

## Abstract

**Abstract:**

**Introduction:**

Childbirth-related perineal trauma (CRPT) is the most common complication of childbirth, affecting 80% of women after a vaginal birth. However, there is a lack of evidence regarding the care of women following CRPT. Specifically, there is a lack of understanding regarding appropriate postnatal CRPT management, wound assessment and treatment of complications. To improve maternal outcomes, this qualitative study aims to explore women’s and healthcare professionals’ (HCPs) views and experiences of current CRPT wound management and healing to understand what they would want from an assessment tool and related care pathway being developed by the Chapter programme of research.

**Methods and analysis:**

A qualitative study guided by an interpretive descriptive approach will be undertaken to explore the views and experiences around CRPT. This will be conducted through individual interviews and focus groups with women (n∼40 participants) who have experienced CRPT within the last 12 months and individual interviews with HCPs (n∼25) who care for women who have experienced CRPT. Supported by specialist interpreters where needed, data collection will be audio recorded and transcribed. Data will initially be analysed using codebook thematic analysis for targeted analysis and then using the Framework Method to facilitate a systematic and flexible exploration of themes within and between groups.

**Ethics and dissemination:**

This study has received ethical approval from the University of Birmingham Science, Technology, Engineering and Mathematical Ethics Review Committee (ERN_23–0666). Supported by a Patient Advisory Group, this study will contribute to the overall outputs and dissemination of the Chapter programme of research, including a core outcome set for trials investigating the care of women experiencing CRPT, the development of a Wound Assessment Tool, professional resources and guidelines for best practice and patient resources. Findings will be disseminated via conference presentations, peer-reviewed publications, the National Institute for Health and Care Research Journals Library, relevant media platforms and plain language summaries, including infographics.

**ISRCTN registration number:**

45172.

**STRENGTHS AND LIMITATIONS OF THIS STUDY**

- Robust data collection will be supported by the use of discussion guides co-designed with the Patient Advisory Group (PAG) comprising women who have experienced childbirth-related perineal trauma (CRPT) and healthcare professionals (HCPs) who care for women who have experienced CRPT.
- Study findings will be strengthened by the diverse sample of women and HCPs that will be recruited for the study.
- Data analysis will be conducted by experienced qualitative health researchers, with data interpretation supported by the PAG and wider multidisciplinary Chapter collaborative group.
- Recruitment and data collection with women who have experienced CRPT in the last 12 months may be challenging due to recall issues, the potential impact of CRPT on their health, and their parenting responsibilities.
- Recruitment of HCPs may be problematic due to the burden of clinical responsibilities on National Health Service professionals.

## Introduction

 Childbirth-related perineal trauma (CRPT) is a vaginal/perineal tear or graze that occurs during vaginal birth. The tears can either occur spontaneously during birth or occur due to a cut (episiotomy) performed by a doctor or midwife to make the opening of the vagina wider to facilitate birth (eg, during an assisted (forceps/ventouse) delivery). CRPT is the most common complication of childbirth, affecting 85% of women after a vaginal birth, with known risk factors for occurrence and severity, including first vaginal birth, Asian ethnicity, operative birth, fetal malposition and birth weight above 4 kg.^1^ Episiotomies make up 26% of all CRPT^2 3^ and are the most frequently performed surgical procedure globally.^4^ In England, 75% of women (450 000 women per annum) require stitches to repair CRPT.^2 5^ Short-term complications, such as infection and dehiscence, are experienced by 6% of women with CRPT, and longer-term consequences, such as incontinence, are reported.^1^

Despite its high prevalence and potential morbidity, research into CRPT management is lacking^4^ and, to date, has focused mainly on trying to prevent CRPT and improving immediate management at the time of repair.^6^ Research related to postnatal management has largely focused on severe tears involving anal sphincter injury, commonly referred to as obstetric anal sphincter injuries,^7^ aiming to reduce complications related to faecal incontinence. For tears not involving the anal sphincter, research has been almost exclusively related to pain management.^8 9^ There is little qualitative or quantitative research related to which women might be more likely to suffer physical complications, the range of long-term health problems experienced and the care and management required.^10^ There is limited dialogue on how, where and when women access follow-on care or treatment for CRPT. Indeed, current literature suggests that there is limited information or support for women who have concerns or complications related to CRPT.^11^

Reviews of women’s experiences of CRPT report various physical and emotional impacts.^11^,^16^ Women report that issues such as pain and incontinence can affect their ability to parent as desired.^11 12^ Women have also reported feeling or being isolated by the impact of CRPT,^11 12^ this being especially true when incontinence is experienced.^13^ Further, psychological impacts^14^ include anxiety around future pregnancies and births.^15 16^ Experiencing CRPT restricts women’s social^11^ and recreational activities, such as exercise,^16^ and has a direct negative effect on their sexual relationships, such as reducing frequency and enjoyment.^12 14 15^

Women in the UK are commonly under midwifery care for the first few postnatal weeks, with support then passing to a health visitor. The routine 6–8 week maternal postnatal check is the responsibility of a woman’s general practitioner (GP), who is also a gatekeeper to appropriate specialist referral.^17^ Evidence of healthcare professional (HCP) views and experiences of CRPT care is limited and narrow in scope, predominantly focussing on midwives,^18^,^20^ with the views and experiences of specialist HCPs who may assess and treat women with complications related to CRPT, such as obstetricians and physiotherapists, largely absent from current qualitative evidence.

There is some qualitative evidence of women’s views and experiences of care for CRPT, with women reporting a lack of consistency of healthcare pathways and variable skills and attitudes of the HCPs working with them.^16^ Women also report being dissatisfied with follow-up care for CRPT, particularly highlighting a lack of information and adequate support.^11 13 15^ The National Institute for Health and Care Excellence (NICE) recommends research to determine what perineal symptoms (particularly pain) should prompt further assessment and management and the development of tools to support the process.^11^ Improvement in CRPT care provision has the potential to alleviate unnecessary worry for women, improve outcomes, increase healthcare efficiency by streamlining services and reduce National Health Service (NHS) costs (eg, each hospital readmission costs the NHS £589, an outpatient visit £172 and a GP visit £64).^21^

The qualitative study presented in the current protocol forms a work package within the wider Chapter research programme, which is funded by the programme grant for Applied Health Research, National Institute for Health and Care Research (NIHR), 202869. The Chapter programme aims to optimise the care of women following CRPT and seeks to address NHS priorities, including ensuring patients receive the right care in the right place, at the right time; providing patients earlier diagnoses and more control over staying well and preventing illness and tackling health inequalities.^22 23^ To address these programme aims, the Chapter includes a range of research, including a quantitative cohort study assessing outcomes for women after CRPT^24^ and a systematic review and analysis of current evidence on CRPT treatment and postnatal management. Furthermore, understanding how current CRPT care is experienced and perspectives on how care could be enhanced to improve maternal outcomes is explored by qualitative enquiry. The present empirical qualitative study aims to explore women’s and HCPs’ views and experiences of CRPT wound management and healing to understand what they would want from a wound assessment tool (WAT) and related care pathways. Objectives for the qualitative study are:

1. To explore the experience of CRPT and postnatal maternity care (relevant to women and HCPs).
2. To identify concerns and worries relating to CRPT (relevant to women and HCPs).
3. To explore and identify outcomes that are important in relation to CRPT (relevant to women and HCPs).
4. To determine what HCPs would want from a WAT, what does a tool look like (eg, time points, delivery method and information required for decision) and find out how they would feel about using a WAT.
5. To investigate what women would want from a care pathway involving a WAT (eg, how and when it is/should be used, what thresholds should prompt a change to the pathway and what care pathways are acceptable to them).
6. To explore HCPs' views on examples of existing WATs, for example: episiotomy healing assessment, Redness, Oedema, Ecchymosis, Discharge, Approximation (REEDA),^25^ Pressure Ulcer Scale for Healing^26^ and Oxford wound healing pathway^27^.
7. To find out what HCPs would want from a care pathway involving a WAT (eg, how they would wish the tool to guide their practice, thresholds for a change in the pathway, where the tools would sit and how they could refer on and escalate care if needed).

## Methods and analysis

### Design

This qualitative study will be guided by an interpretive descriptive (ID) approach.^28^ ID is aligned with a constructivist and naturalistic orientation to inquiry,^29^ the purpose of which is to capture themes and patterns within subjective perceptions and generate an interpretive account capable of informing clinical understanding and decision-making.^28^

### Setting

This study will run across the UK to help facilitate diversity in the sample of women and HCPs, with a view to the findings being credible and transferable.

### Eligibility criteria

#### Women

1. Inclusion criteria: cis women, aged 18 years and above, residents in the UK, have experienced CRPT in the last 12 months and speak English or one of the six most spoken languages in healthcare settings in the West Midlands (eg, Romanian, Urdu, Bengali, Polish, Arabic and/or Hindi).
2. Exclusion criteria: transgender men and inability to provide consent.

Inclusion criteria rationale: the time limit around a diagnosis of CRPT in the last 12 months will help to facilitate recall of ‘recent’ experiences and the participation of women who may have struggled to participate in the earlier postnatal months and provide insight into the potential long-term impacts of CRPT. Loss of the infant will not exclude a woman from participation, as the exclusion of bereaved women could undermine their identity as a parent and direct experience of CRPT. For some, participation in research following bereavement can be meaningful and can be handled sensitively and reflect the participant’s needs.^30^ We have purposively chosen not to include transgender men in the study, although we recognise that this may be exclusionary. However, transgender men may experience complications exacerbated by hormonal and physical changes that are beyond the scope of our current study.

#### Healthcare professionals (HCPs)

1. Inclusion criteria: participants will have to be NHS-registered HCPs, aged 18 years and above, currently involved in the provision of care in the NHS to women in the perinatal period.
2. Exclusion criteria: inability to consent, student (eg, none registered) HCPs*.*

Inclusion criteria rationale: including qualified professionals working with women across the perinatal period (prenatal, antenatal, intrapartum and postnatal) will ensure a broad and comprehensive exploration of CRPT and wound management.

### Sampling and recruitment

Purposive sampling will be used to facilitate the realisation of diversity among the participants such that the study findings are transferable. Snowballing^31^ will also be used to allow participants to use their networks to provide a rich context for the study and further facilitate potential diversity.

To reflect the diversity of the maternal population, we aim to include women with a range of different demographic characteristics related to region (urban/rural), ethnicity, first language, index of multiple deprivations ((IMD) in England only)/socioeconomic status, body mass index (BMI) and smoker/non-smoker. Likewise, to capture the range of maternal experiences of care, we will aim to include a range of CRPT characteristics related to time since childbirth (≤6 months or 7–12 months), type of CRPT, parity, follow-up care accessed at the time of delivery, mode of delivery (unassisted/assisted) and CRPT complication (breakdown/infection/revision required).

Pathways for recruiting women have been informed by discussions with the Chapter programme Patient Advisory Group (PAG). Women will be recruited via social media (eg, but not limited to, Twitter, Facebook, Instagram and Snapchat). We will also target recruitment via parent (mother) and baby groups across the West Midlands (as this is where the research team is based). Such parent and baby groups will be asked to advertise and promote the study via their own channels on our behalf, and we may attend sessions in person to directly recruit eligible women. Various appropriate organisations, such as charities and community organisations (eg, The MASIC Foundation which supports women who have suffered serious injuries during childbirth) will also advertise the research on our behalf.

Where recruitment of women is face to face (eg, in mother and baby groups), potential participants will be provided with written and verbal information about the study by members of staff and/or the research team. They will also be given the contact details of the research team to pass on to any other potential participants (eg, snowballing). Participants who self-identify via adverts and social media will be encouraged to contact the research team directly if they wish to know more and/or participate. More detailed information will then be sent (eg, participant information leaflet) via email, phone or social media interaction. Participant facing materials, including consent forms, will be available in English and a further six of the most spoken languages in healthcare settings in the West Midlands (eg, Romanian, Urdu, Bengali, Polish, Arabic and/or Hindi).

We aim to include HCPs with a range of characteristics related to type of clinician, years qualified, community/primary/secondary/tertiary care provider, region within which they provide care (urban/rural), IMD quintile of practice (in England) and clinician gender. HCPs will be identified via the research teams’ personal and professional networks, social media advertising and promotion via appropriate Royal colleges. Study information (eg, participant information leaflet) will be sent to HCPs as part of the direct approach from the research team.

Reasonable steps will be taken to ascertain that participants are genuine women or HCPs with experience of CRPT, such as verifying contact details, including a UK address or telephone number, checking HCPs’ names against professional registers, meeting the participant face-to-face or on video prior to data collection, checking email contacts (to review the time zones sent from) and providing thank-you vouchers that are redeemable in UK-only stores.^32^

### Anticipated sample sizes

We anticipate up to 40 women participants who have sustained any form of CRPT and up to 25 HCPs who are providing CRPT care to women. Numbers will remain flexible to ensure that the overall sample and associated data have sufficient information power to develop new knowledge in relation to the research objectives.^33^

### Consent

Written informed consent will be sought wherever possible. In cases where the study-related paperwork has not been received, is not fully completed or there are issues around English or digital literacy, then we will seek alternative forms of informed consent, including electronic (eg, returning a scan or photo of the consent form) or verbal (eg, where the consent form will be read out in full and audio recorded at the start of the interview or focus group (FG) in a different audio file to that of the data collection). Informed consent (including written, electronic and/or verbal) will gain permission for agreement to participate, demographic data collection, audio-recorded dialogue of discussion and anonymised data sharing for further research. At the beginning of each audio recording, participants will be asked to verbally (re)confirm consent.

The participant information leaflet will clearly state that participants are able to withdraw from the project at any time without needing to justify the reasons and how they need to do this (eg, by contacting the research team directly). If they choose to withdraw within 2 weeks of the data collection event, they will be informed that their data, where possible (as this can be challenging in the context of a group discussion), will be securely destroyed. If they choose to withdraw after 2 weeks, then they will be informed that their anonymised data will still be used in the project, as their information will already have been incorporated into the ongoing analysis. The consent form requires participants to confirm that they have understood that they are able to withdraw. FG and interview facilitators will reiterate the terms of withdrawal before the start of data collection.

Women and HCPs will be offered vouchers as an appreciation of their time (£25 for an interview and £40 for FG participation). Participants who choose to withdraw before the data collection event (eg, interview or FG) will not be offered any compensation. Participants who contribute to data collection will be compensated, even if they subsequently withdraw. Provision is available to cover reasonable childcare and travel expenses for women if this supports participation.

### Data collection

Discussion guides (online supplemental file 1 (women) and online supplemental file 2 (HCPs)) will be used to support data collection and have been developed using prior literature, wider study objectives, discussions with the research team, feedback from the PAG and piloting guides with an eligible HCP and woman.

Aligned with the preferences expressed by our PAG, individual interviews and FGs will be used to facilitate data collection with women. Given the sensitive nature of the subject and to ensure an inclusive approach, we will offer women the choice of their preferred data collection method (eg, face-to-face, by telephone or via a video call). We will use trained specialist interpreters to help facilitate participation in up to six different languages to aid diversity in the sample and to ensure that the voices of women, where English is not their first language, can be heard.

We will use semistructured interviews (face-to-face, by telephone or via a video conferencing platform such as Zoom or Teams) to facilitate data collection with HCPs (eg, but not limited to, community and hospital midwives, health visitors, physiotherapists, GPs and obstetricians).

We anticipate that we will use a blended approach of face-to-face and virtual data collection to facilitate choice and to maximise the facilitation of many interviews and FGs over a short time. There is growing evidence that rapport can be readily established using remote techniques and that the flexibility and adaptability afforded by technology minimise inconvenience and disruption to participants.^34 35^ This may be particularly helpful for HCPs with busy schedules and women who need to provide care for their child(ren). We will also ensure that women (and HCPs if needed) are supported to participate, for example, by sending out guidance and offering a trial run with a researcher to use the data collection platform.

Participants will be asked to complete a short demographic questionnaire to facilitate sampling and description of the participating sample. For women, this will include questions on location, age, first language, gender identity, sexual orientation, ethnicity, education, smoking status, BMI, type of birth and circumstances of the CRPT. For HCPs, this will include questions on location, age, gender identity, sexual orientation, ethnicity, clinician role and frequency of providing CRPT care. Following advice from the PAG, we added information for participants as to why the collection of this data is important for understanding the study sample and to inform transferability. Interviews and FGs will be audio recorded and transcribed clean verbatim by an external specialist transcription company that is compliant with the General Data Protection Regulations (GDPR). We will quality assure the interpretation in non-English data collection events by having the same question and associated participant responses (approximately 5–10 min) in each language transcribed, translated and checked. The remainder of the interview or FG will only have the English parts of the discussion transcribed. Non-English speaking participant views will be interpreted into English by the professional interpreter during the interview or FGs. Data collection and initial analysis will take place iteratively,^36^ with data collection continuing until the research team judges that the data and sample have sufficient depth and breadth to address the study’s aim.^33^ Data collection and analysis will be conducted by proficient qualitative health researchers who have experience working with women and HCPs regarding sensitive health topics.

### Data analysis

The initial targeted analysis will use codebook thematic analysis to inform subsequent work packages within the Chapter programme looking at WAT development and outcome measures. Codebook thematic analysis is a method for effectively identifying relevant data for each domain of interest. Such domains have been identified from prior literature, research objectives and familiarisation of the data in question.^37^ Further experiential data analysis will then be informed by the Framework Method (FM)^38 39^ to facilitate a systematic and flexible approach to facilitate exploration of themes and patterns within and between the groups of interest. The FM is not aligned with a particular epistemological, philosophical or theoretical stance (ie, it is a-theoretical); rather, it is a flexible tool that facilitates the generation of themes.^38^ It can, therefore, be adapted for use with ID methodology as ID aims to identify thematic patterns and commonalities, as well as account for individual variation in experience within the interpretive account. Data management will be supported by the use of NVivo software and inductive analysis will be guided by the seven stages of FM proposed by Gale *et al*^38^: (1) transcription, (2) familiarisation, (3) coding, (4) development of a working analytical framework, (5) application of the analytical framework, (6) charting data in the framework matrix and (7) interpretation of data.^40^

This study aims to support the development of a CRPT WAT and related care pathway, both of which will have to meet the needs of a diverse range of women as service users and HCPs as care providers. Data will be analysed for women and HCPs as collective groups; no subanalysis based on sample characteristics is planned. However, in line with good practice for presenting thematic findings, any clear differences within the sample will be highlighted in the study write up.

### Patient and public involvement

The Chapter programme PAG, comprising 11 women who have experienced CRPT, has been established. It has met quarterly since its inception, interspersed with specific individual work tasks. The PAG has been actively involved in designing and writing study documentation for the present qualitative study. For example, the group advised on the design of recruitment adverts for the study by providing a critical review of all participant-facing materials, including the addition of signposts to support women experiencing distress. They helped to co-produce the discussion guides and demographic questionnaires, ensuring the study, which is in a sensitive topic area, is acceptable to a diverse range of women.

The PAG will continue to provide guidance on recruitment, acceptability and problem-solving support. As the project progresses, they will provide invaluable insight into the interpretation of study data and help share findings. We have captured the impact our PAG has made on the Chapter study using the public involvement and in research impact toolkit (PIRIT) toolkit.^41^

### Data management

The University of Birmingham is the nominated sponsor and data controller of this study. All members of the research team will comply with the principles of the UK GDPR 2018 and maintain up-to-date Good Clinical Practice training. The study will adhere to the relevant data protection policies of the University of Birmingham. All data collected on paper, including consent forms, will be stored securely in a locked cabinet in a locked room only accessible to the research team and relevant regulatory authorities. All data collected in electronic format will be stored in secure networks or encrypted password-protected computers. All data will be held securely in the custody of the Chapter study work package/programme leads for a minimum of 10 years after publication of the main Chapter programme results in accordance with the University of Birmingham Research Data Management Policy.

Confidentiality will be assured throughout and after the project except for instances of participant disclosure of the risk of harm or where there is a need for safeguarding intervention. Participants will be informed of this via the participant information leaflet and will be provided with an opportunity to discuss this prior to participation. The research team, where appropriate, will also try to discuss the need to break confidentiality if disclosure of harm occurs. The translation and transcribing companies will be subject to a confidentiality/personal data transfer agreement. Confidentiality will be maintained in all outputs of the project, including publications. Quotes used in publications will use the unique study ID or culturally appropriate pseudonyms. The identifier key will be stored separately from identifiable data.

Data collected from participants who later decide to withdraw their contributions within 2 weeks of the data collection will be securely destroyed (where possible). An individual interview will be deleted from the audio recorder/remote platform. For FGs, where it may be difficult to delete the audio responses of an individual participant who has withdrawn, the participant’s contributions will be redacted on the transcript and will not be considered for analysis.

### Risks

Given the potentially sensitive issues that may emerge in discussions, our experienced qualitative researchers will undertake data collection guided by Dempsey *et al’s* framework of essential elements for conducting qualitative research.^42^ Distressing topics will be handled sensitively, and the study-specific distress pathway will be followed,^43^ including signposting to additional support as appropriate. Professional language interpreters used by the study will receive specific training from the research team (potentially with support from the PAG) related to CRPT, study processes, including consent, and how the interviews and FGs need to be interpreted with a focus on conceptual equivalence rather than word-for-word or literal translation.^44^ A debrief between the researcher(s) and the interpreter will be held after each interview or FG to identify any interpretation issues, as appropriate. Where practicable, we will try to use only a small pool of interpreters to support efficacy.

## Supplementary material

10.1136/bmjopen-2024-088248 online supplemental file 1

10.1136/bmjopen-2024-088248 online supplemental file 2
