# Supplementary material for: Exploring views and experiences of childbirth-related perineal trauma: a qualitative study protocol for developing a wound management tool and care pathway
Source: BMJ Open. 2025 Apr 25;15(4):e088248. doi: 10.1136/bmjopen-2024-088248 (PMC12035485; doi:10.1136/bmjopen-2024-088248)
Supplement: online supplemental file 1 [file bmjopen-15-4-s001.docx]

**Interview/discussion group guide: Women affected by cuts and/or tears during childbirth**

| **Study Objective** | **Key Discussion Points** |
| --- | --- |
| 1. To explore the experience of CRPT and maternity care postnatally (secondary and primary care) for women and involvement of HCPs | - Explore experience of receiving care relating to CRPT  - Explore knowledge and terminology around CRPT  - Explore understanding around cause and effect (physical, mental, emotional, social) of CRPT |
| 2. To explore care pathways experienced by women affected by CRPT | - Explore care pathways that women affected by CRPT have experienced  - Explore the views of women on care pathways |
| 3. To identify concerns and worries relating to CRPT | - Explore challenges experienced relating to CRPT  - Explore attitudes associated with CRPT |
| 4. To find out the outcomes that are important to women | - Explore the outcomes that are important to women around care of CRPT |
| 5. To investigate what women would want from a care pathway involving a WAT? | - Explore what women affected by CRPT would want from a care pathway integrated with an assessment tool |
