# Supplementary material for: Exploring views and experiences of childbirth-related perineal trauma: a qualitative study protocol for developing a wound management tool and care pathway
Source: BMJ Open. 2025 Apr 25;15(4):e088248. doi: 10.1136/bmjopen-2024-088248 (PMC12035485; doi:10.1136/bmjopen-2024-088248)
Supplement: online supplemental file 2 [file bmjopen-15-4-s002.docx]

**Interview discussion guide: Healthcare Professionals**

| **Objective** | **Key Discussion Points** |
| --- | --- |
| 1. To explore the experience of CRPT and maternity care postnatally (secondary and primary care) for women and involvement of HCPs | - Explore experience of providing care relating to CRPT  - Explore knowledge and terminology around CRPT |
| 2. To explore care pathways utilised by HCPs to address CRPT | - Explore attitudes associated with CRPT  - Explore care pathways that HCPs use  - Explore the views of HCPs on care pathways |
| 3. To identify concerns and worries relating to CRPT | - Explore understanding around cause and effect (physical, mental, emotional, social) of CRPT  - Explore challenges experienced relating to CRPT |
| 4. To find out the outcomes that are important to HCPs | - Explore the outcomes that are important to HCPs around care for women with CRPT |
| 5. To explore HCPs views on examples of existing WATs | - Explore views of HCPs on existing WATs such as Episiotomy healing assessment: Redness, Oedema, Ecchymosis, Discharge, Approximation (REEDA), Pressure Ulcer Scale for Healing (PUSH), Oxford Wound healing pathway. |
| 6. To determine what HCPs would want from a WAT, what a tool might look like and how they would feel about a WAT | - Explore what HCPs would want from an assessment tool  - Explore their feelings about an assessment tool |
| 7. To find out what HCPs would want from a care pathway involving a WAT | - Explore what HCPs would want from a care pathway integrated with an assessment tool |
