# Supplementary material for: Exploring views and experiences of childbirth-related perineal trauma: a qualitative study protocol for developing a wound management tool and care pathway
Source: BMJ Open. 2025 Apr 25;15(4):e088248. doi: 10.1136/bmjopen-2024-088248 (PMC12035485; doi:10.1136/bmjopen-2024-088248)
Supplement: Supplementary file 3 [file bmjopen-2024-088248.reviewer_comments.pdf]

PEER REVIEW HISTORY

BMJ Open publishes all reviews undertaken for accepted manuscripts. Reviewers are asked to complete a checklist review form and are provided with free text boxes to elaborate on their assessment. These free text comments are reproduced below.

ARTICLE DETAILS

Title (Provisional)

Exploring Views and Experiences of Childbirth-Related Perineal Trauma: A Qualitative Study Protocol for Developing a Wound Management Tool and Care Pathway

Authors

Jones, Laura; Delicate, Amy; Waigwa, Susan; Hodgetts Morton, Victoria; Morris, R. K.; Whitehurst, Jane; hillman, sarah; Collaborative Group, Chapter

VERSION 1 - REVIEW

**Reviewer**	**1**
**Name**	**Kurnaz, Döndü**
**Affiliation**	**Marmara Universitesi**
**Date**	**19-Jan-2025**
**COI**	**None**

Title:

The title can be shortened, for instance: "Exploring Views and Experiences of Childbirth-Related Perineal Trauma: A Qualitative Study Protocol for Developing a Wound Management Tool and Care Pathway."

Abstract:

The background section only discusses evidence. However, evidence cannot be derived from a qualitative study. There should be a background sentence aligned with the study's objective. For example: "The views and experiences regarding CRPT wound management and healing are... (Source...)."

Introduction:

The problem/phenomenon being studied is well-defined, and its significance is clearly stated. It is known among those working in the field that this problem represents a gap in the literature. However, to avoid confusion for those not directly involved in the specific area, the rationale for conducting a qualitative study, rather than a quantitative one, should be explained more clearly. What will we see from a qualitative study that we wouldn’t observe in a quantitative one? This distinction needs to be clarified.

A comprehensive set of research questions appropriate to the study's aims and objectives has been developed. The objectives are highly professional and well-prepared.

Methods:

The inclusion criteria and their justification are well explained. However, in a study on Childbirth-Related Perineal Trauma (CRPT), confounding factors such as the degree of perineal trauma, the number of births, instrumental delivery, the presence of diabetes (which may affect wound healing), and the baby's weight should be reconsidered. These should be added as exclusion criteria, as they could significantly influence the results. Although it is mentioned that these data will be collected in the sample, no method for isolating these factors in the analysis is described. Excluding these criteria as much as possible would lead to more reliable results.

The sample of healthcare personnel to be included in the study is quite diverse. Given the aim of developing a measurement tool, this could be an appropriate sample selection. However, the analysis should be done both collectively and within each specific group of healthcare professionals.

The timing of the study, considering when the women are most ready and memory factors, will enhance the quality of the research.

The experience of the researchers conducting the study and analysis in qualitative research should be mentioned.

Data processing and data analysis are sufficiently explained. Issues related to data credibility and techniques to enhance reliability are well covered.

The quality and reliability of the study data largely depend on the study design and methodology. Therefore, I believe these pre-prepared protocols will serve as a guide for well-designed studies. They will also help save time for the researchers.

Reviewer 2

Name East, Christine

Affiliation La Trobe University, School of Nursing and Midwifery / Judith Lumley Centre

Date 18-Mar-2025

COI None

Thank you for the opportunity to review this protocol for a qualitative study: "Exploring women’s and healthcare professionals’ views an experiences of childbirth related perineal trauma to inform the development of a wound management tool and care pathway: protocol for a qualitative study." The topic is timely and of great relevance to women and healthcare professionals as we work together to improve childbirth and its sequelae. The protocol establishes the rationale for the study and proposes an appropriate means to consider the views of both consumers and clinicians. As such, the protocol provides confidence that the study will be rigourously undertaken. I look forward to hearing of the findings in due course.

VERSION 1 - AUTHOR RESPONSE

Reviewer: 1

Dr. Döndü Kurnaz, Marmara Universitesi

Comments to the Author:

Title: The title can be shortened, for instance: "Exploring Views and Experiences of Childbirth-Related Perineal Trauma: A Qualitative Study Protocol for Developing a Wound Management Tool and Care Pathway."

Thank you for this suggestion. The title has been shortened in line with your comment.

Abstract: The background section only discusses evidence. However, evidence cannot be derived from a qualitative study. There should be a background sentence aligned with the study's objective. For example: "The views and experiences regarding CRPT wound management and healing are... (Source...)."

Thank you. The introduction of the abstract has been re-written to make the purpose and scope of the qualitative study clear.

Introduction: The problem/phenomenon being studied is well-defined, and its significance is clearly stated. It is known among those working in the field that this problem represents a gap in the literature. However, to avoid confusion for those not directly involved in the specific area, the rationale for conducting a qualitative study, rather than a quantitative one, should be explained more clearly. What will we see from a qualitative study that we wouldn't observe in a quantitative one? This distinction needs to be clarified. A comprehensive set of research questions appropriate to the study's aims and objectives has been developed. The objectives are highly professional and well-prepared.

Thank you. Chapter is a programme of work on the topic of childbirth-related perineal trauma of which the present qualitative study is one work package. For example, Chapter also has a cohort study which is looking at quantitative outcomes. We have made the context of the wider Chapter programme clearer and addressed your comments about rationale for the qualitative study at page 4 of the revised manuscript.

Methods: The inclusion criteria and their justification are well explained. However, in a study on Childbirth Related Perineal Trauma (CRPT), confounding factors such as the degree of

perineal trauma, the number of births, instrumental delivery, the presence of diabetes (which may affect wound healing), and the baby's weight should be reconsidered. These should be added as exclusion criteria, as they could significantly influence the results. Although it is mentioned that these data will be collected in the sample, no method for isolating these factors in the analysis is described. Excluding these criteria as much as possible would lead to more reliable results.

Thank you for this interesting feedback. The Chapter quantitative cohort study will be looking at maternal outcomes and analysis will adjust for confounding factors. The presented qualitative study aims to look at women's experiences of care after CRPT and perceived effect on outcomes. As such the risk factors for sustaining CRPT, or experiencing complications are inconsequential; the qualitative work looks to understand how care is experienced when the injury has occurred, not why care is needed. The aim for diversity in the sample (such as type of tear and parity) is to enable data to be collected that represents the range of experiences in the maternal population and related needs and wants for care. Clarifying text to this effect has been added at page 5.

The sample of healthcare personnel to be included in the study is quite diverse. Given the aim of developing a measurement tool, this could be an appropriate sample selection. However, the analysis should be done both collectively and within each specific group of healthcare professionals.

Thank you for this insight. The range of healthcare professionals is diverse as this reflects the reality of postnatal care in the UK and the range of health care professionals a women may encounter following CRPT. Also, the wound assessment tool that Chapter will be developing is to be used universally by all perinatal healthcare professionals irrespective of role, experience level, or healthcare setting. Therefore, we do not require the granular details of what a hospital midwife wants in a wound assessment tool as separate to a community health visitor; the tool must work for all of them as a single product. Text to clarify these issues has been added at page 9.

The timing of the study, considering when the women are most ready and memory factors, will enhance the quality of the research. The experience of the researchers conducting the study and analysis in qualitative research should be mentioned.

Thank you, the experience of the researchers conducting the study have been added at page 8.

Data processing and data analysis are sufficiently explained. Issues related to data credibility and techniques to enhance reliability are well covered. The quality and reliability of the study data largely depend on the study design and methodology. Therefore, I believe these pre-prepared protocols will serve as a guide for well-designed studies. They will also help save time for the researchers.

Thank you for reviewing the manuscript and for your considered comments which have enhanced the revised protocol.

Reviewer: 2

Dr. Christine East, La Trobe University

Comments to the Author:

Thank you for the opportunity to review this protocol for a qualitative study: "Exploring women's and healthcare professionals' views an experiences of childbirth related perineal trauma to inform the development of a wound management tool and care pathway: protocol for a qualitative study." The topic is timely and of great relevance to women and healthcare professionals as we work together to improve childbirth and its sequelae. The protocol establishes the rationale for the study and proposes an appropriate means to consider the views of both consumers and clinicians. As such, the protocol provides confidence that the study will be rigorously undertaken. I look forward to hearing of the findings in due course.

Thank you for reviewing the protocol and for your considered comments.
